# Evaluation of Neutrophil Gelatinase-Associated Lipocalin and Cystatin C in Early Diagnosis of Chronic Kidney Disease in the Absence of the Gold Standard

**DOI:** 10.31661/gmj.v9i0.1698

**Published:** 2020-06-22

**Authors:** Fatemeh Masaebi, Mehdi Azizmohammad Looha, Zhuoyu. Wang, Elaheh Zarean, Malihe Nasiri, Faranak Kazerouni, Fatemeh Gharishvandi, Farid Zayeri

**Affiliations:** ^1^Department of Biostatistics, Faculty of Paramedical Sciences, Shahid Beheshti University of Medical Sciences, Tehran, Iran; ^2^Zhuoyu Wang, Academy of Medical Engineering and Translational Medicine, Tianjin University; ^3^Modeling in Health Research Center, Shahrekord University of Medical Sciences, Shahrekord, Iran; ^4^Faculty of Nursing and Midwifery, Shahid Beheshti University of Medical Sciences, Tehran, Iran; ^5^Department of Laboratory Medicine, School of Allied Medicine, Shahid Beheshti University of Medical Sciences, Tehran, Iran; ^6^Department of Clinical Biochemistry, Faculty of Medicine, Tehran University of Medical Sciences, Tehran, Iran; ^7^Proteomics Research Center and Department of Biostatistics, Faculty of Paramedical Sciences, Shahid Beheshti University of Medical Sciences, Tehran, Iran

**Keywords:** Chronic Kidney Diseases, Neutrophil Gelatinase-Associated Lipocalin, Cystatin C, Bayesian Approach, Latent Class Model, Sensitivity, Specificity

## Abstract

**Background:**

Glomerular filtration rate (GFR) is considered as a gold standard of kidney function. However, using GFR as the gold standard is not common in clinical practice, because its direct measurement is usually expensive, cumbersome, and invasive. In the present study, we assessed the predictive power of two other biomarkers, Cystatin-C (Cys-C) and Neutrophil Gelatinase-Associated Lipocalin (NGAL) for early detection of chronic kidney diseases (CKD) in the absence of a gold standard.

**Materials and Methods:**

In this study, 72 patients who referred to the Shohadaye Tajrish Hospital of Tehran, Iran, for measuring their kidney function were studied. The ELISA method was utilized for measuring plasma NGAL (PNGAL) and serum Cys-C (SCys-C). The Bayesian latent class modeling approach was applied to asses the predictive power of these biomarkers.

**Results:**

While both the biomarkers had rather high sensitivities (PNGAL=91%, SCys-C= 89%), the specificity of SCys-C biomarker was very lower than the one of PNGAL (SCys-C=56%, PNGAL=94%). The estimated area under the receiver operating characteristic (ROC) curve for SCys-C as the single biomarker for the diagnosis of CKD was about 0.76, while a similar estimate for PNGAL was 0.93. The added value of PNGAL to SCys-C for the diagnosis of CKD in terms of the ROC curve was about 0.19, while the added value of SCys-C to PNGAL was less than 0.02.

**Conclusion:**

In general, our findings suggest that PNGAL can be utilized as a single reliable biomarker for early detection of CKD. In addition, results showed that when a perfect gold standard is not available, Bayesian approaches to latent class models could lead to more precise sensitivity and specificity estimates of imperfect tests.

## Introduction

Chronic kidney disease (CKD) is one of the most prevalent problems of the urinary tract system. This problem is usually defined as abnormalities of kidney structure of function present for more than three months [1]. CKD in its early stage is a silent problem and its symptoms are evident when patients reached end-stage renal failures [2]. Late detection of CKD causes incidence of other subsequent outcomes of the disease [3]. The incidence, prevalence, and global burden of CKD are increasing rapidly; recent reports show that the incidence rate of this disease in many parts of the world was over 200 per million individuals in 2012 [4], and the global prevalence rate is 14.3% in the most population [5]. CKD was ranked as 27th among causes of death in 1990, while it became as 18th and 12th in 2010 and 2015, respectively [6]. Regarding increasing prevalence rate of CKD and its considerable burden throughout the world, early diagnostic and proper treatment of this disease could help physicians to control the progress and improve the consequences of the disease. In this context, identifying related indicators of CKD is of great importance for diagnosing this disease in its early stage. In the past decades, researchers have developed a variety of markers for evaluating kidney function. Among these markers, the glomerular filtration rate (GFR) is known as the most optimal index of kidney function because of its reduction after structural kidney damage. In CKD patients, it is also known that many other kidney functions decline in parallel with GFR [7,8]. According to 2009 Kidney Disease: Improving Global Outcomes (KDIGO) clinical practice guideline, the GFR of 90mL/min/1.73 represents healthy kidneys and the value less than 15 mL/min/1.73 indicates the end stage of kidney disease [9]. The GFR less than 60 mL/min/1.73 for more than three months is the common threshold of CKD [10]. Since direct measurement of GFR is impossible, some alternatives are instead utilized to measure the clearance of exogenous substances that eliminated by filtration only [11]. In this context, inulin clearance is widely used as a gold standard method to measure GFR [12]. However, the rigorous measurement of inulin clearance is invasive, cumbersome, and expensive because it needs continuous intravenous infusion and repeated assessments of blood and urine [13]. Another common method for measuring GFR is estimating its value using the formulas developed by the Modification of Diet in Renal Disease (MDRD) study group [14]. According to the MDRD, although, the eGFR is usually an acceptable approximation, like other creatinine-based approximations of GFR, it may lead to underestimated or overestimated results, for example in some ages or body mass index (BMI) groups [15]. Besides, among most commonly-used formulas, one of them was able to estimate the GFR using serum creatinine, age, race, and sex. In addition, another version of the MDRD formula applied six variables for estimating GFR. Despite all we have mentioned above, since limited accuracy and significant misclassification have been seen in the estimation of parameters, the formulas cannot be considered as a proper method [16-18]. Because of problems with using creatinine-based estimates of GFR, researchers have recently developed some other alternatives for estimating GFR. Cystatin –C (Cys-C) is one of these alternatives that is widely used as an indicator of kidney function [19]. Cys-C level in urine represents the overall function of kidney, so that an increase in its concentration is related to a reduction in GFR. Unlike creatinine, Cys-C is independent of diet and muscle mass. These characteristic makes Cys-C a sensitive and popular indicator for estimating GFR [20]. Neutrophil Gelatinase- Associated Lipocalin (NGAL), also called lipocalin-2, is another well- known biomarker that is frequently reported in the published literature as an early indicator of chronic kidney damage [21,22]. This protein is expressed in neutrophils and low levels of it could be found in kidney, prostate, and alimentary and respiratory tracts [23]. In recent years, the predictive power of NGAL for early detection of kidney disease is explored in numerous studies [24-26]. In addition, some studies compared predictive power of NGAL with other biomarkers like Cys-C and creatinine, for early diagnosis of different renal diseases [26-28]. As mentioned before, several researchers have previously studied the predictive power of Cys-C and NGAL biomarkers in the early detection of CKD. In the published literature, controversial findings have been reported about the sensitivity and specificity of the described biomarkers. In the present study, we follow two important objects. First, we aim to determine the predictive power of Cys-C and NGAL biomarkers in the early detection of CKD using the common indices, i.e. sensitivity and specificity. Second, we estimate the added value of each biomarker to the other one. In other words, we aim to know whether adding the information from each biomarker to the other one leads to a significant increase in the accuracy of diagnosing CKD. To achieve these goals, we used a latent class model in the absence of the gold standard [29].

## Materials and Methods

###  Study Population

 In this cross-sectional study, patients who referred to the Shohadaye Tajrish hospital (affiliated to Shahid Beheshti University, Tehran, Iran) for examining their kidney function were assayed. The Characteristics of the patients was shown in Table-1.

###  Inclusion and Exclusion Criteria

 In this study, only patients with normal urea and creatinine were recruited. Patients with chronic cardiovascular and liver disease were not included in the study. The written study consent was obtained from each patient.

###  Data Collection

 In this study, 10cc fasting blood sample was collected from each participant. Then, 5cc of this sample was used in tubes containing EDTA and plasma was immediately separated using a refrigerated centrifuge. The rest 5cc blood was collected in tubes without anticoagulant. After 30 minutes, the coagulated blood was centrifuged to obtain the serum. The maintained serum and plasma were stored at -200c until the test day. The ELISA method was used for plasma NGAL (PNGAL) and serum Cys-c (SCys-c) measurements.

###  Ethical Statements

 This research has been approved by the Ethics Committee of the Shahid Beheshti University in Tehran, Iran (the ethical code: IR.SBMU.RETECH.REC.1397.1182).

###  Statistical Analysis

 In recent decades, a variety of statistical tools have been proposed by the data analysis for assessing the predictive power of multiple tests (biomarkers) for diagnosing disease with no gold standard. Among these methods, the Bayesian latent class models (BLCMs) interesting approaches, which enable researches to combine the results of multiple tests (considering the conditional independence between tests) and predict the diagnostic power of these tests, in the absence of reliable gold standards. For two diagnostic tests, with dichotomous (binary) results, the latent class models can be written as:

p(T_1_+,T_2_+)=Π(sens_1_sens_2_)+(1-Π)(1-spec_1_)(1-spec_2_)

p(T_1_-,T_2_+)=Π(sens_2_(1-sens_1_))+(1-Π)(spec_1_(1-spec_2 _))

p(T_1_-,T_2_-)=Π(1-sens_1_)(1-sens_2_)+(1-Π)(spec_1 _spec_2_)

p(T_1_+,T_2_-)=Π(sens_1_(1-sens_2_))+(1-Π)(spec_2_(1-spec_1_))

where Tj(j=1,2), sensj,specj and Π show jth test, the sensitivity of jth test, specificity of jth test and disease prevalence, respectively. One of the most important aspects of this LCM is that when we apply model there is no need to consider any test as the gold standard and we can combine some imperfect diagnostic tests for obtaining unbiased estimators [29]. In this study, based on a non-parametric method, the amount of area under the receiver operating characteristic (ROC) curve (AUC) was calculated for, and then (as a new test) was added to the model in the presence of and the AUC was calculated. Finally, the difference between these two amounts showed that using can define the amount of the diagnostic accuracy increase. Also, Statistical analyses were performed using the WinBUGS for Windows, Version 1.4.3 that was developed by the MRC Biostatistics Unit, at the University of Cambridge, United Kingdom and R 3.4.1 software.

## Results

 In this study, 72 patients (24 male and 48 female) who referred to the Shohadaye Tajrish hospital were investigated. The mean age of these patients was 54.50±8.06, ranged from 40 to 70 years. Table-1 shows the descriptive statistics for some characteristics of these patients. The mean of SCys-C and PNGAL for these patients was 423.76±744.97 and 78.73±105.44, respectively. According to the cut-off point value of 98 for SCys-C, 42 individuals (58.3%) had CKD. While, regarding the cut-off point value of 32 for PNGAL, 25 persons (34.7%) were diagnosed as patients with CKD. Among these 72 patients, 24 persons (33.3%) had SCys-C value higher than 98 and PNGAL value higher than 32, concurrently (Table-2). In the next step of data analysis, we used two BLCMs. In model 1, we assessed the added value of PNGAL (as the new test) to the SCys-C (as the current test) and in model 2 we evaluated the added value of SCys-C (as the new test) to the PNGAL (as the current test). Table-2 shows the obtained estimates from fitting these models. According to the estimates in Table-2, one can conclude that while both the biomarkers had rather high sensitivity (91% for PNGAL v.s 89% for SCys-C), the specificity of SCys-C biomarker was much lower than PNGAL (56% v.s 94%). Regarding the estimated AUCs from two models, some other interesting findings can be obtained. The estimated AUC based on SCys-C as the single biomarker for diagnosing CKD was about 76%, while a similar estimate for PNGAL was 93%. It seems PNGAL can classify CKD more accurately than SCys-C. Furthermore, the estimated AUC difference form two models tell us that the added value of PNGAL to SCys-C for diagnosing CKD was about 19%, while the added value of SCys-C to PNGAL was less than 2%.

## Discussion

CKD, as an important risk indicator of cardiovascular disease, is a major health problem throughout the world. The prevalence and incidence estimate of this disease is rather vague because it is commonly undetectable and asymptomatic in the early stages. The diagnosis of CKD in the later stages imposes a high economic cost to the health systems of all world countries annually. Regarding this global health burden, identifying relevant biomarkers for detecting CKD in the early stages is of great importance. In this research, we assessed the power of two well-known biomarkers for early diagnosis of CKD [30]. In previous decades, creatinine has been widely used as a common marker for detecting CKD. However, its late response to changes in the structure and function of the kidney encouraged researchers to identify other biomarkers such as Cys-C for early diagnostic of CKD [19]. In this context, Yilmaz *et al*. conducted a study on 26 patients with renal failure (stage 1-4) in order to assess the role of Cys-C in diagnosing CKD. Their results demonstrated that Cys-C is an appropriate marker for estimating GFR with high accuracy (senseivity=92%, specificity=1) and detecting CKD in the early stages [31]. In the current study, we found that Cys-C has low power for detection of non-CKD patients (specificity=56%). It should be noted that high sensitivity and specificity in the study by Yilmaz *et al*. might not be precisely indicated the accuracy of the Cys-C marker, because the measurement error was not eliminated in their gold standard (eGFR) [18]. This could affect the sensitivity and specificity of Cys-C in their study. In addition, the aforementioned error in the gold standard may affect the correct diagnosis of the best cut-point for Cys-C. The results obtained in our study were not in accordance with what they have achieved. This may be due to absence of gold standard in our work. On the other hand, the sample size used in the Yilmaz *et al*. study seems not to be adequate which makes the reported Sensitivity and specificity more questionable. NGAL has recently been recognized as another appropriate marker for early diagnosis of CKD [24-26]. Increasing serum and urine levels of NGAL after kidney damage could be considered as an eligible marker for the early diagnosis of kidney damage [32,33]. Moreover, the results of several related studies reported a significant correlation between NGAL, Cys-C, and GFR. Seems there is an agreement about the NGAL as a proper marker for early CKD detection [34,35]. Basturk *et al*. used eGFR as the gold standard for early detection of CKD on 45 patients in stage 1 and reported a sensitivity and specificity of 72.2% by using an optimal cut-point value of 98.71 ng/mL for NGAL. In our study, the sensitivity and specificity of NGAL for diagnosing CKD were 91% and 94%, respectively. This notable difference may be attributed to the error in the eGFR results that were used as the gold standard in the Basturk *et al*. study. In Another conducted by Bolignano *et al*. on 96 white European patients with various degrees of the renal disease, the eGFR was considered as the gold standard and the results showed that the accuracy of NGAL (sensitivity=83.9% and specificity=53.8%) for detecting CKD was notably lower than what we estimated in our study [35]. One reason for this difference might be the presence of error measurement in the gold standard used in the Bolignano *et al*. study. This means that the use of inaccurate gold standard might be led to misleading estimates for sensitivity and specificity of a marker in the detection and diagnosis. In other words, estimating the sensitivity and specificity in the absence of a gold standard may lead to more accurate results than using a gold standard with possible measurement error. Similar to our work, some researchers have previously compared the accuracy of NGAL and Cys-C in the early detection of CKD. In a study by Mitsnefes *et al*. in 2007 on 45 CKD patients aged 6 to 21 years, the obtained results showed that adding NGAL to Cys-C might increase the diagnostic accuracy by using GFR as the gold standard [34]. In another study, Gharishvandi *et al*. used the cut point of 78mL/min/1.73 for eGFR as the gold standard and examined the diagnostic power of these two markers. Their findings indicate that PNGAL with sensitivity =96% and specificity=100% could have higher diagnostic power compared to Cys-C (sensitivity of 92% and specificity of 60 %) [25]. We found a high degree of consistency in our results with Mitsnefes *et al*. and Gharisvandi *et al*. studies although the actual amount of GFR and its estimated value were applied in their studies respectively. Moreover, based on our results, it is noticeable that the NGAL marker was more powerful compared to Cys-C and also had higher accuracy to evaluate the presence of CKD. Furthermore, NGAL improved the diagnostic power of CKD when the Cys-C used simultaneously as a marker.

In our literature review, we found no study about assessing the diagnostic power of NGAL and Cys-C in the absence of gold standard using advanced statistical models. As mentioned before, no reliable gold standard is available for CKD and markers like eGFR have their own errors [18]. One of the great advantages of the current study was the use of the BLCMs method for evaluating the diagnosis power of the described biomarkers. These statistical models enable us to find unbiased estimates for the model parameters in the absence of the gold standard. Using these advanced approaches also enables us to estimate the added value of a biomarker to the other. Another advantage of this method is using the Bayesian framework for estimating the model parameters. The Bayesian estimates are not sensitive to small sample size. This helpful advantage of Bayesian estimations could handle the most important problem of such datasets (inadequate sample size) which can be seen in similar studies in this field. On the other hand, our research has some limitations too. Due to a lack of financial supports, the ELISA method was used to measure the Cys-C and NGAL markers. This limitation might cause a lower degree of precision compared to some other methods such as Luminescence Safety Assay.

## Conclusion

 In the present study, our findings showed that while both the Cys-C and NGAL biomarkers had acceptable sensitivity for early detection of CKD patients, the specificity of NGAL was much higher than the Cys-C marker. In addition, our modeling approach revealed that a precise and reliable gold standard is not available–for instance, in early diagnosing of CKD– using statistical methods with no need for gold standard could provide accurate findings in the term of the sensitivity and specificity of the markers. In general, regarding satisfactory diagnostic power of NGAL, the use of this marker in the early detection of CKD is recommended.

## Conflict of Interest

 There is no conflict of interest.

**Table 1:** Characteristics of the Patients

**Characteristics**	**Minimum**	**Maximim**	**Mean**	**Standard Deviation**
**Age**	40.00	70.00	54.50	8.06
**Weight**	49.00	113.00	79.28	13.65
**Creatinine**	0.70	1.40	1.02	0.17
**Urea**	11.00	45.00	27.10	8.40
**Fibrinogen**	146.00	345.00	215.80	66.40

**Table 2:** Accuracy of SCys-C and PNGAL for Diagnosing CKD Using BLCM

**Accuracy Index**	**Model 1**	**Model 2**
PNGAL Sensitivity	0.91(0.87,0.95 )*	0.91(0.87,0.95)
PNGAL Specificit **y**	0.94(0.90,0.9 **8)**	0.94(0.90,0.98)
SCys-C Sensitivity	0.89(0.85,0.9 **3)**	0.89(0.85,0.93)
SCys-C Specificity	0.56(0.5,0.6 **2)**	0.56(0.5,0.62)
SCys-C A **UC**	0.76 (0.74,0.78)	-----
PNGAL AUC	-- **---**	0.93(0.89,0.97)
SCys-C & NGAL AUC	0.95(0.92,0.9 **8)**	0.95(0.92,0.98)
AUC Difference	0.19^**^(0.17,0.21)	0.02^***^(0.004,0.04)

* (95% CI) **Added value of PNGAL to Cys-C ***Added Value of Cys-C to NGAL
